# Vitamin D3 suppresses the cholesterol homeostasis pathway in patient‐derived glioma cell lines

**DOI:** 10.1002/2211-5463.13679

**Published:** 2023-07-31

**Authors:** Ran Yuan, Wei Zhang, Yong‐Ping You, Gang Cui, Zhengliang Gao, Xiuxing Wang, Jian Chen

**Affiliations:** ^1^ Institute of Functional Nano & Soft Materials (FUNSOM) Soochow University Suzhou China; ^2^ Chinese Institute for Brain Research Beijing China; ^3^ Research Unit of Medical Neurobiology Chinese Academy of Medical Sciences Beijing China; ^4^ Department of Neurosurgery, Beijing Tiantan Hospital Capital Medical University Beijing China; ^5^ China National Clinical Research Center for Neurological Diseases, Beijing Tiantan Hospital Capital Medical University Beijing China; ^6^ Neuropathology, Beijing Neurosurgical Institute Capital Medical University Beijing China; ^7^ Department of Neurosurgery, The First Affiliated Hospital of Nanjing Medical University, Institute for Brain Tumors, Jiangsu Collaborative Innovation Center for Cancer Personalized Medicine Nanjing Medical University China; ^8^ Department of Neurosurgery & Brain and Nerve Research Laboratory The First Affiliated Hospital of Soochow University Suzhou China; ^9^ Fundamental Research Center, Shanghai YangZhi Rehabilitation Hospital (Shanghai Sunshine Rehabilitation Center), School of Medicine Tongji University Shanghai China; ^10^ Institute of Geriatrics (Shanghai University), Affiliated Nantong Hospital of Shanghai University (The Sixth People's Hospital of Nantong), School of Medicine Shanghai University Nantong China; ^11^ National Health Commission Key Laboratory of Antibody Techniques, Department of Cell Biology, Jiangsu Provincial Key Laboratory of Human Functional Genomics, School of Basic Medical Sciences Nanjing Medical University China

**Keywords:** calcitriol, cholesterol homeostasis, glioblastoma, vitamin D receptor, vitamin D3

## Abstract

Glioblastoma is one of the most common malignant brain tumors. Vitamin D, primarily its hormonally active form calcitriol, has been reported to have anti‐cancer activity. In the present study, we used patient‐derived glioma cell lines to examine the effect of vitamin D3 and calcitriol on glioblastoma. Surprisingly, vitamin D3 showed a more significant inhibitory effect than calcitriol on cell viability and proliferation. Vitamin D receptor (VDR) mediates most of the cellular effects of vitamin D, and thus we examined the expression level and function of VDR via gene silencing and gene knockout experiments. We observed that VDR does not affect the sensitivity of patient‐derived glioma cell lines to vitamin D3, and the gene encoding VDR is not essential for growth of patient‐derived glioma cell lines. RNA sequencing data analysis and sterolomics analysis revealed that vitamin D3 inhibits cholesterol synthesis and cholesterol homeostasis by inhibiting the expression level of 7‐dehydrocholesterol reductase, which leads to the accumulation of 7‐dehydrocholesterol and other sterol intermediates. In conclusion, our results suggest that vitamin D3, rather than calcitriol, inhibits growth of patient‐derived glioma cell lines via inhibition of the cholesterol homeostasis pathway.

## Graphical abstract

Vitamin D3 exerts a significantly stronger inhibitory effect on cell viability and cell proliferation of patient‐derived glioma cell lines than calcitriol, and this effect is mediated through inhibition of cholesterol homeostasis and cholesterol synthesis.

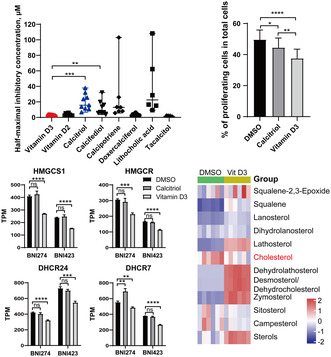

**Abbreviations**
7‐DHC: 7‐dehydrocholesterol
Cas9: CRISPR‐associated protein 9
CRISPR: clustered regularly interspaced short palindromic repeats
CNS: central nervous system
CYP24A1: cytochrome P450 family 24 subfamily A member 1
CYP27A1: cytochrome P450 family 27 subfamily A member 1
CYP27B1: cytochrome P450 family 27 subfamily B member 1
DHCR24: 24‐dehydrocholesterol reductase
DHCR7: 7‐dehydrocholesterol reductase
EdU: 5‐ethynyl‐2′‐deoxyuridine
GBM: glioblastoma
GSC: glioma stem cells
HMGCR: 3‐hydroxy‐3‐methylglutaryl‐CoA reductase
HMGCS1: 3‐hydroxy‐3‐methylglutaryl‐CoA synthase 1
IC_50_
: half‐maximal inhibitory concentration
PCSK9: proprotein convertase subtilisin/kexin type 9
sgRNA: single guide RNA
shRNA: small hairpin RNA
SRE: sterol‐regulatory element
TCGA: The Cancer Genome Atlas
VDR: vitamin D receptors

Glioma, a type of central nervous system (CNS) tumor, is considered to derive from neuroglial stem or progenitor cells [1]. More than half of glioma cases are glioblastoma (GBM) [2], which is one of the most malignant CNS tumors and is defined as grade 4 by the World Health Organization [3]. Despite the development of potential therapeutic approaches, there has been no significant improvement in GBM patient survival in recent years [4]. In glioma, tumor cells with self‐renewal and differentiation ability are referred to as glioma stem cells (GSC) [5, 6, 7, 8]. GSC were considered the driving force of tumor growth and heterogeneity, and one of the reasons for treatment resistance and high recurrence rate [9, 10]. Therefore, GSC were one of the main *in vitro* models for exploring glioma. We used GBM cell lines for research as well, which were isolated from GBM patient tumor samples and cultured with the serum‐free stem cell culture method. These cell lines were called patient‐derived glioma cell lines. Patient‐derived glioma cell lines have similar characteristics to GSC and retain a certain proportion of genomic and transcriptome characteristics from patients.

Vitamin D is a group of fat‐soluble steroids, the most important of which are vitamin D3 (cholecalciferol) and vitamin D2 (ergocalciferol). Vitamin D from the diet or synthesized from the skin is not active and requires hydroxylation mainly through these enzymes, such as cytochrome P450 family 2 subfamily R member 1 (*CYP2R1*), cytochrome P450 family 27 subfamily A member 1 (*CYP27A1*) and cytochrome P450 family 27 subfamily B member 1 (*CYP27B1*), to form active vitamin D (calcitriol), which then binds to vitamin D receptors (VDR) and regulates downstream genes [11, 12]. VDR mediates most of the cellular effects of vitamin D, such as cell growth, immune function, inflammation and even nervous system regulation [13, 14, 15, 16]. Clinical studies have shown that vitamin D deficiency may contribute to an increased risk of CNS disease. For GBM, there was an inverse correlation between the level of calcifediol [25(OH)D3, a metabolite of vitamin D] in serum and the risk of GBM [17]. Furthermore, the expression of *VDR* was correlated with the prognosis of GBM patients [18, 19]. Therefore, some studies were interested in the anti‐cancer effect of vitamin D on GBM. As early as the end of the last century, studies reported that calcitriol and calcifediol can significantly inhibit the growth of GBM cell lines *in vitro* [20, 21, 22]. The main manifestations were inhibition of cell proliferation, reduction of migration rate and reduction of the stemness of glioma stem‐like cells [23]. These effects were related to the activation of the metabolism of phosphor sphingolipids [24, 25, 26] or the activation of cytochrome P450 family 24 subfamily A member 1 (*CYP24A1*) [27]. In addition, vitamin D analogs tacalcitol and calcipotriol also exhibited similar anti‐cancer effects [26, 28].

The studies of vitamin D in GBM were virtually focused on the active form of vitamin D, calcitriol. However, vitamin D3 has significant inhibitory effects on patient‐derived glioma cell lines as well. Therefore, we hypothesized that there are differences in the mechanism of anti‐cancer effects between vitamin D3 and calcitriol. To verify this hypothesis, the effects of vitamin D3 and calcitriol on cell viability, proliferation, and apoptosis were determined. The changes in gene expression in patient‐derived glioma cell lines after drug treatment were analyzed by RNA sequencing. The results revealed that cholesterol homeostasis was the most significantly down‐regulated pathway after vitamin D3 treatment, but calcitriol did not. Meanwhile, from sterolomics analysis, the treatment of vitamin D3 led to the excess accumulation of 7‐dehydrocholesterol (7‐DHC) and other sterol intermediates. Notably, 7‐DHC is a common precursor of vitamin D3 and cholesterol [29] and the mRNA expression level of 7‐dehydrocholesterol reductase (*DHCR7*) that plays a key role in the process of 7‐DHC to cholesterol [29] decreased after vitamin D3 treatment. This suggested that *DHCR7* was a key target that vitamin D3 affected cholesterol homeostasis.

## Materials and methods

### Patient‐derived glioma cell lines and cell culture

Glioma cell lines were derived from patients with stage 4 GBM. BNI274, BNI423, BNI17 and BNI7‐11 were obtained from WZ. G709 and GNJ755 were obtained from Y‐PY. G98 was obtained from GC. GBM17 was obtained from ZG. GSC11 was obtained from XW. [30]. Informed written consent and approval of the institutional review board of Beijing Tiantan Hospital Affiliated to Capital Medical University was obtained (KY2014‐021‐02) and the study complies with the guidelines set by the Declaration of Helsinki. All patient‐derived glioma cell lines were cultured in Dulbecco's modified Eagle's medium/F12 (Gibco, Waltham, MA, USA), 1% penicillin/streptomycin (Solarbio, Beijing, China), 1X B‐27 without vitamin A supplement (Thermo Scientific, Waltham, MA, USA), 1X N‐2 supplement (Thermo Scientific), 0.5% glutaMAX‐1 (Gibco), 5 mm Hepes (Aladdin, Shanghai, China), 600 μg·mL^−1^ glucose (Sangon, Shanghai, China), 50 μm 2‐mercaptoethanol (Sigma‐Aldrich, Burlington, MA, USA), 20 ng·mL^−1^ EGF (Novoprotein, Wuhan, China) and 20 ng·mL^−1^ bFGF (OriGene, Rockville, MD, USA) and placed within a 37 °C, 5% CO_2_, 5% O_2_ and 90% humidity sterile incubator [31].

### Chemical compounds

Almost all compounds were purchased from Selleck (Shanghai, China), including vitamin D3 (S4063), vitamin D2 (S4035), calcitriol (S1466), calcifediol (S1469), calcipotriene (S3739), doxercalciferol (S1467), lithocholic acid (S4003), lovastatin (S2061) and cholesterol (S4154). Tacalcitol (HY‐32337) was purchased from MedChemExpress (Shanghai, China). These compounds were added to the cell culture medium at the concentrations indicated, as appropriate. If not labeled, the concentrations of vitamin D3, calcifediol and calcitriol used for BNI274 were 5 μm, and for BNI423 were 2.5 μm.

### Cell viability assay

All tested cells were seeded in 96‐well plates in triplicate with 1500 cells per well. Compounds were added into the cell culture medium for 72 h of treatment. Cell viability was measured with Cell Titer‐Glo Luminescent Cell Viability Assay kit (Promega, Madison, WI, USA) in accordance with the manufacturer's instructions.

### Apoptosis assay

For annexin V staining, BNI274 or BNI423 of 20–30% confluent was seeded in six‐well plates. After overnight incubation, cells were treated with vitamin D3, calcitriol, or vehicle (dimethylsulfoxide) at the concentrations shown, as appropriate, for 36 h. Staining was performed using a FITC Annexin V Apoptosis Detection Kit with PI (BioLegend, San Diego, CA, USA). Flow cytometry was performed on Becton Dickinson LSR Fortessa flow cytometer (BD Biosciences, San Jose, CA, USA) and data were analyzed with flowjo software (TreeStar, San Francisco, CA, USA).

### 5‐Ethynyl‐2′‐deoxyuridine (EdU) incorporation assay

BNI274 or BNI423 was seeded 20–30% confluent in 6‐well plates, and compounds were added into cell culture medium at the concentrations shown in the figure legends for 36‐h treatment. After incubation with 10 μm EdU for 2 h, the cells were fixed in cold 4% paraformaldehyde and performed staining using BeyoClick EdU Cell Proliferation Kit with Alexa Fluor 488 (Beyotime, Beijing, China) in accordance with the manufacturer's instructions. Axio Observer (ZEISS, Oberkochen, Germany) was used to acquire images and the number of EdU‐positive cells was counted manually.

### Lentivirus packaging

Lentivirus was prepared by co‐transfecting HEK293T cells with Lentiviral expression plasmids : psPAX2 : pMD2.G in a 5 : 3 : 2 ratio using polyethyleneimine Linear (MW25,000). Here, psPAX2, pMD2.G, and lentiGuide‐Puro were obtained from AddGene (Cambridge, MA, USA). Supernatants that contained lentivirus particles were collected twice between 36 and 72 h, and lentivirus particles were concentrated by centrifugation with PEG‐6000 (Sangon). Before use, lentivirus particles were resuspended in the serum‐free defined medium and filtered through 0.22 μm Millex® sterile syringe filters (Millipore, Burlington, MA, USA).

### Small hairpin RNA (shRNA) and single guide RNA (sgRNA)

For the knockdown of *VDR*, the shRNA sequence targeting *VDR* was generated in the lentiviral pLKO.1‐Puro vector (AddGene). The sequences of shRNA were:

shVDR_6542: CCGGCCTCCAGTTCGTGTGAATGATCTCGAGATCATTCACACGAACTGGAGGTTTTTG
shVDR_6543: CCGGCTCCTGCCTACTCACGATAAACTCGAGTTTATCGTGAGTAGGCAGGAGTTTTTG
shVDR_6544: CCGGTTGGCTTTGCTAAGATGATACCTCGAGGTATCATCTTAGCAAAGCCAATTTTTG

For the knockout of gene *VDR*, the gRNA was inserted into lentiGuide‐Puro vector with green or red fluorescent protein. The CRISPR‐associated protein 9 (Cas9)‐target sites were:

VDR‐site‐1: ATTCACCTGCCCCTTCAACG
VDR‐site‐2: CCATCATTCACACGAACTGG
VDR‐site‐3: TGACAGATGAGGAAGTGCAG
LacZ‐site: CCCGAATCTCTATCGTGCGG
PSMD1‐site: ACCAGAGCCACAATAAGCCA

### Cell infection

For lentiviral infection, 1–2 × 10^5^ Patient‐derived glioma cell lines were seeded in 6‐well plates with 1.2 mL medium supplemented, then high titer lentivirus and protamine sulfate (10 μg·mL^−1^) were added. After 4–6 h of incubation, the supernatant was replaced with fresh medium. Selection with antibiotics was started 36–24 h after infection with puromycin (2 μg·mL^−1^, Selleck) or blasticidin (50 μg·mL^−1^, Solarbio). Monoclonal cell lines with bilateral allelic knockouts were obtained by limiting dilution and flow sorting with Becton Dickinson FACSAria Fusion flow cytometer (BD Biosciences). The efficiency of knockout efficiency was verified by Western blots and Sanger sequencing. Sanger sequencing was performed by GENEWIZ (Suzhou, China). The sequences of primers for PCR were:

VDR‐site‐3‐Forward 5′‐AGTGCTTCTCCTCTGGACCG‐3′
VDR‐site‐3‐Reverse 5′‐GAGGGAGCCCCGAGTGTTA‐3′

### Clustered regularly interspaced short palindromic repeats (CRISPR) competition assay

CRISPR competition assay was used to evaluate gene essentiality. Briefly, patient‐derived glioma cell lines were infected with green‐fluorescent‐protein‐target‐sgRNA (*VDR*, *PSMD1* as a positive control, and *LacZ* as a negative control) or red‐fluorescent‐protein‐sgLacZ (as negative control), then mixed cells with different fluorescent in equal proportions. The ratios of cells with different fluorescent were measured on Day 3, Day 7, and Day 14 with Becton Dickinson LSR Fortessa flow cytometer (BD Biosciences), and data were analyzed with flowjo (TreeStar).

### RNA sequencing analysis

Total RNA was isolated using Trizol reagent extraction in accordance with the manufacturer's instructions. For sequencing, more than 2 μg of RNA of each sample was sent to GENEWIZ (Suzhou, China) for library preparation and RNA sequencing. For statistical analysis of RNA sequencing data, read counts were generated using featureCount and normalized using DESeq2 in r [32, 33]. In a pairwise DE comparison between vitamin D3‐treated and control, significant DE is filtered based on |log_2_‐fold change| > 1 and adjusted *P* < 0.05. These data are heatmapped by ComplexHeatmap to show the top 100 down‐regulated genes [34]. Gene set enrichment analysis for hallmark pathways was calculated via fgsea in r [35].

### RNA extraction and real‐time PCR

Total RNA was isolated from cells using Trizol reagent extraction in accordance with the manufacturer's instructions. Then, 0.5–1 μg total RNA was used to prepare cDNA using a PrimeScript RT reagent Kit (TaKaRa Bio, San Jose, CA, USA). Real‐time PCR was performed with 2X SYBR Green qPCR Master Mix (Bimake, Shanghai, China) on a CFX96 RealTime System (Bio‐Rad, Hercules, CA, USA). RNA expression levels were normalized to co‐amplified GAPDH. The real‐time primers were:

VDR‐Forward 5′‐TCTCCAATCTGGATCTGAGTGAA‐3′
VDR‐Reverse 5′‐GGATGCTGTAACTGACCAGGT‐3′
PCSK9‐Forward 5′‐ATGGTCACCGACTTCGAGAAT‐3′
PCSK9‐Reverse 5′‐GTGCCATGACTGTCACACTTG‐3′
VGLL4‐Forward 5′‐AACTGCAACCTCTCGCACTG‐3′
VGLL4‐Reverse 5′‐GCTCGGGCTCCTTGTAATTCT‐3′
HMGCS1‐Forward 5′‐CATTAGACCGCTGCTATTCTGTC‐3′
HMGCS1‐Reverse 5′‐TTCAGCAACATCCGAGCTAGA‐3′
HMGCR‐Forward 5′‐TGATTGACCTTTCCAGAGCAAG‐3′
HMGCR‐Reverse 5′‐CTAAAATTGCCATTCCACGAGC‐3′
GLI1‐Forward 5′‐AGCGTGAGCCTGAATCTGTG‐3′
GLI1‐Reverse 5′‐CAGCATGTACTGGGCTTTGAA‐3′
GLI2‐Forward 5′‐CCCCTACCGATTGACATGCG‐3′
GLI2‐Reverse 5′‐GAAAGCCGGATCAAGGAGATG‐3′
SMO‐Forward 5′‐CTGTCCTGCGTCATCATCTTT‐3′
SMO‐Reverse 5′‐CCACAGCAAGGATTGCCAC‐3′
PTCH1‐Forward 5′‐CCAGAAAGTATATGCACTGGCA‐3′
PTCH1‐Reverse 5′‐GTGCTCGTACATTTGCTTGGG‐3′
CYP24A1‐Forward 5′‐GATTTTCCGCATGAAGTTGGGT‐3′
CYP24A1‐Reverse 5′‐CCTTCCACGGTTTGATCTCCA‐3′
GAPDH‐Forward 5′‐AATCCCATCACCATCTTCCA‐3′
GAPDH‐Reverse 5′‐TGGACTCCACGACGTACTCA‐3′

### Luciferase assay

BNI274 or BNI423 was infected the sterol‐regulatory element (SRE)‐luciferase reporter by lentivirus. Cells were seeded in 96‐well plates in triplicate with 3000 cells per well. Compounds were added into the cell culture medium at the concentrations shown, as appropriate. After incubation for 24 h, the luciferase reporter assay was performed using a Bright‐Glo Luciferase Assay System (Promega) in accordance with the manufacturer's instructions. The luciferase activity was normalized to cell viability. The SRE‐luciferase reporter was provided by GENEWIZ.

### Cholesterol detection assay

BNI274 was seeded in 96‐well plates in triplicate with 3000 cells per well. Vitamin D3 with the concentration of 5 μm or dimethylsulfoxide was added into the cell culture medium for 24‐h treatment. Then total cellular cholesterol level was measured using Cholesterol/Cholesterol Ester‐Glo Assay (Promega) in accordance with the manufacturer's instructions. The detection result was normalized to cell viability.

### Sterolomics analysis

BNI274 were harvested after 24 h of treatment of 5 μm vitamin D3, 5 μm calcifediol, 5 μm calcitriol or dimethylsulfoxide. Each treatment was performed in six replicates, and the number of cells per sample was exceeded 5 million. Samples were sent to LipidALL Technologies (Changzhou, China) for lipid extraction and related analysis. Sterolomics analysis was conducted at LipidALL Technologies as previously described [36]. Lipids were extracted from cells using a modified version of the Bligh and Dyer's protocol [37]. Lipid extract was resuspended in 500 μL of 1 n ethanolic potassium hydroxide containing 5 μg of butylated hydroxytoluene. An internal standard cocktail (50 μL) comprising d_6_‐lanosterol, d_6_‐zymosterol, d_7_‐lathosterol, d_7_‐7‐dehydrocholesterol, d_6_‐sitosterol and d_6_‐cholesterol (Avanti Polar Lipids, Alabaster, AL, USA) was added to the samples. The samples were incubated at 225 ***g*** for 1 h at 37 °C. At the end of incubation, 250 μL of MilliQ water (Merck Millipore, Burlington, MA, USA) and 1 mL of *n*‐hexane were added. The samples were mixed thoroughly by vortexing, and centrifuged at 13400 ***g*** for 5 min 4 °C. Clear upper phase containing total oxysterols and sterols in hexane was transferred to a new tube. The extraction was repeated once with another 1 mL of *n*‐hexane. The pooled extract was dried in a SpeedVac (Eppendorf, Hamburg, Germany) under organic mode. Oxysterols and sterols were derivatised to obtain their picolinic acid esters prior to LC/MS analysis on a U3000 DGLC system (Thermo Fisher, Waltham, MA, USA) coupled to a QTRAP 6500 Plus system (Sciex) and quantitated by referencing to the spiked internal standards as previously described [36].

### Western blots and antibodies

Cultured cells were lysed with RIPA buffer (Beyotime) with phenylmethanesulfonyl fluoride (Beyotime) and protease inhibitor cocktail (Bimake). The protein concentration of each sample was measured with a BCA Protein Assay Kit (Thermo Scientific). An equal amount of protein of each sample was loaded on PAGE gel by electrophoresis to separate the target protein and then transferred to the poly (vinylidene difluoride) membrane (Merck Millipore). Blots were incubated with primary antibodies overnight at 4 °C and followed by horseradish peroxidase‐linked antibodies at room temperature for 1 h. Antibodies purchased from Cell Signaling Technology (Danvers, MA, USA) included vitamin D3 receptor (D2K6W, 12550S), anti‐mouse lgG (7076S) and snti‐rabbit lgG (7074S) and those purchased from OriGene included GAPDH (TA802519).

### Statistical analysis

All other statistical comparisons were carried out with two‐sided Student's tests. *P* < 0.05 was considered statistically significant.

## Results

### Patient‐derived glioma cell lines are sensitive to vitamin D3

**Fig. 1:**
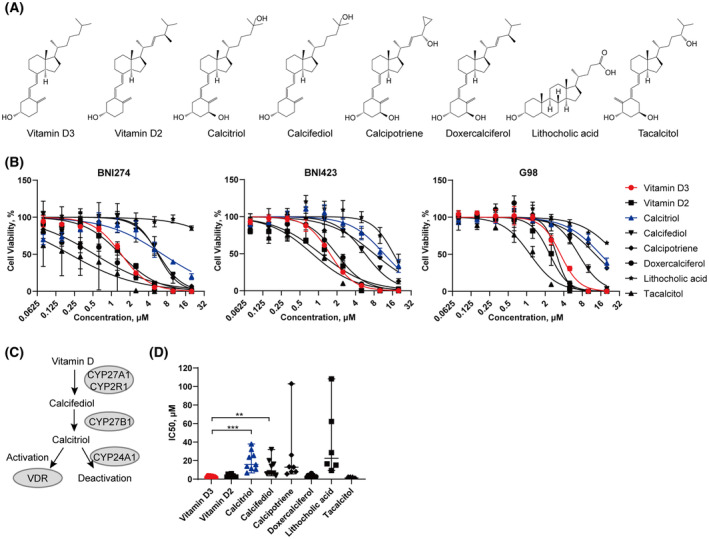
Inhibitory effects of vitamin D3 and its analogues on patient‐derived glioma cell lines. (A) The structure of vitamin D3 and its analogues. (B) Cell viability assays for BNI274, BNI423 and G98 exposed to 0‐20 μm vitamin D3 and its analogues after 72 h of treatment. (C) A simplified summary of metabolic processes of vitamin D3. (D) A summary of IC_50_ for vitamin D3 and its analogues in different patient‐derived glioma cell lines. Median with range, *n* = 9. Values over 110 μm were not shown. **P < 0.01 and ***P < 0.001 according to Student's *t*‐test.

To more comprehensively examine the effects of vitamin D on patient‐derived glioma cell lines, eight small molecule compounds of vitamin D and their analogs were selected (Fig. 1A). Vitamin D3 and vitamin D2 are the most important members of the vitamin D family. Vitamin D3 *in vivo* is usually hydroxylated to calcifediol (25(OH)D3) mainly by CYP2R1 and CYP27A1 and then hydroxylated to the hormonally active form of vitamin D, calcitriol, mainly by CYP27B1 (Fig. 1C). CYP24A1 is the key enzyme for calcitriol inactivation [38]. Apart from the above‐mentioned, tacalcitol is a synthetic vitamin D3 analogue, doxercalciferol is a synthetic vitamin D2 analogue and calcipotriene is a synthetic derivative of calcitriol [28, 40]. Lithocholic acid was also added in as an activator of VDR.

Here, we examined the inhibitory effects of these vitamin D and its analogues on nine patient‐derived glioma cell lines. Among them, the drug dose curves of three representative cell lines to these compounds are shown (Fig. 1B) and the half‐maximal inhibitory concentration (IC_50_) calculated by prism (GraphPad Software Inc., San Diego, CA, USA) is also shown (Fig. 1D and Table 1). The results indicated that the patient‐derived glioma cell lines being examined were sensitive to vitamin D3 and vitamin D2. Meanwhile, it was worth noting that the IC_50_ values of vitamin D3, vitamin D2 and their synthetic analogues were significantly lower than that of the active form of vitamin D (vitamin D3 vs. calcitriol, *P* = 0.0002) and its precursor (vitamin D3 vs. calcifediol, *P* = 0.0060). The IC_50_ of Calcitriol's synthetic derivatives calcipotriene (median = 13.14 μm) and VDR activator lithocholic acid (median = 45.43 μm) were also higher. Such evidence supports the hypothesis that the inhibitory effects of vitamin D3 and vitamin D2 on patient‐derived glioma cell lines were not obtained through *VDR*. Therefore, verification experiments for this hypothesis were carried out. Because vitamin D3 is the form of vitamin D that humans consume from the diet to a greater extent than vitamin D2, the subsequent experiments mainly used vitamin D3 as a representative.

**Table 1:** Half‐maximal inhibitory concentration (IC_50_) of vitamin D and its analogues on nine cell lines of patient‐derived glioma cell lines.

IC_50_ (μm)	Vitamin D3	Vitamin D2	Calcitriol	Calcifediol	Calcipotriene	Doxercalciferol	Lithocholic acid	Tacalcitol
BNI274	1.444	1.407	6.798	5.843	5.658	0.5305	108.2	0.2124
G98	3.536	2.368	15.77	6.945	12.94	3.041	28.6	1.132
BNI423	1.534	1.085	11.59	5.714	8.025	1.947	16.52	0.7917
BNI17	1.45	3.44	10.61	3.645	8.017	1.921	9.318	0.6737
BNI7.11	2.579	2.218	13.87	6.687	13.34	3.587	15.14	1.385
GSC11	3.215	2.697	37.91	16.21	26.08	3.302	> 110	2.328
GNJ755	1.851	3.351	23.66	13.91	102.9	2.612	> 110	2.493
GBM17	3.709	5.318	32.09	20	> 110	4.031	62.26	1.87
G709	3.236	5.953	25.58	32.07	> 110	6.101	> 110	2.54

### Vitamin D3 inhibited the cell proliferation of patient‐derived glioma cell lines

**Fig. 2:**
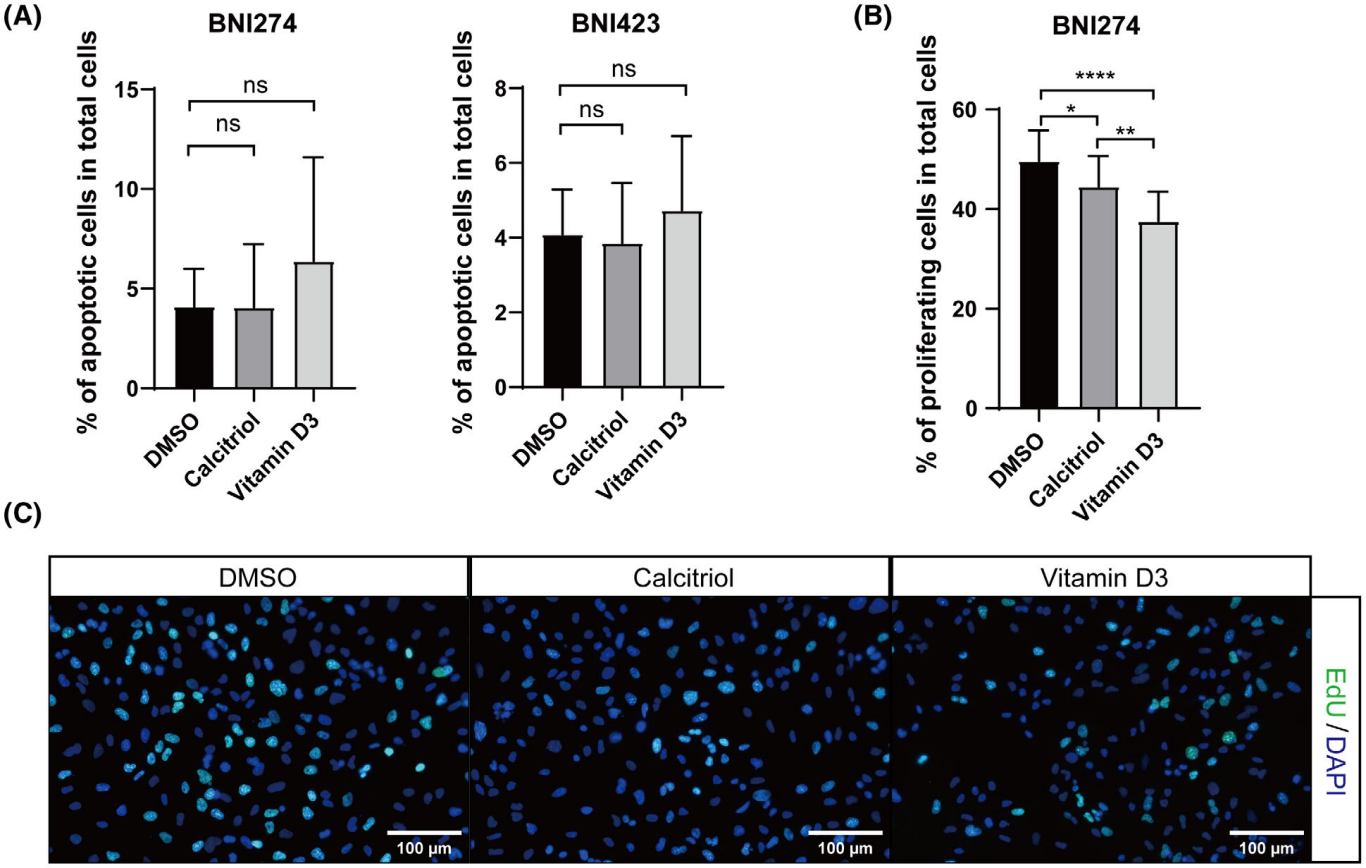
Vitamin D3 inhibited cell proliferation of patient‐derived glioma cell lines. (A) Percentage of apoptotic cells in BNI274 and BNI423 after 36 h of treatment with calcitriol or vitamin D3 evaluated by annexin V/PI staining assays. *n* = 3; mean ± SD. (B, C) Quantification (B) and representative images (C) of proliferating cells in BNI274 after 36 h of treatment with calcitriol or vitamin D3 evaluated by an EdU incorporation assay. Scale bar = 100 μm. The total number of cells quantitated was > 2500 and the number of images quantitated was 16 in each group. The concentration of vitamin D3 or calcitriol used was 5 μm for BNI274 and 2.5 μm for BNI423. Data are the mean ± SD, Student's *t*‐test: *P < 0.05, **P < 0.01, ****P < 0.0001. ns, not significant.

To explore how cell viability was inhibited, cell apoptosis and cell proliferation assays were performed for BNI274 or BNI423 treated with vitamin D3 or calcitriol for 36 h. For the apoptosis assay, the number of apoptotic cells was counted by an annexin V/PI assay. The result showed that the percentage of apoptotic cells tended to increase under vitamin D3 treatment (Fig. 2A), although it was not statistically significant (BNI274: *P* = 0.3580, BNI423: *P* = 0.2949). For the proliferation assay, the proliferative ability of cells was quantified by EdU labeling of proliferating cells (Fig. 2B,C). The percentage of proliferating cells in the vitamin D3‐treated group decreased significantly compared to the control group (*P* < 0.0001). The percentage of proliferating cells in the calcitriol‐treated group also decreased, but not as significantly as in the vitamin D3‐treated group (calcitriol vs. vitamin D3: *P* = 0.0028).

### *VDR* is not an essential gene for patient‐derived glioma cell lines, nor does it affect the inhibitory effect of vitamin D3 on patient‐derived glioma cell lines

**Fig. 3:**
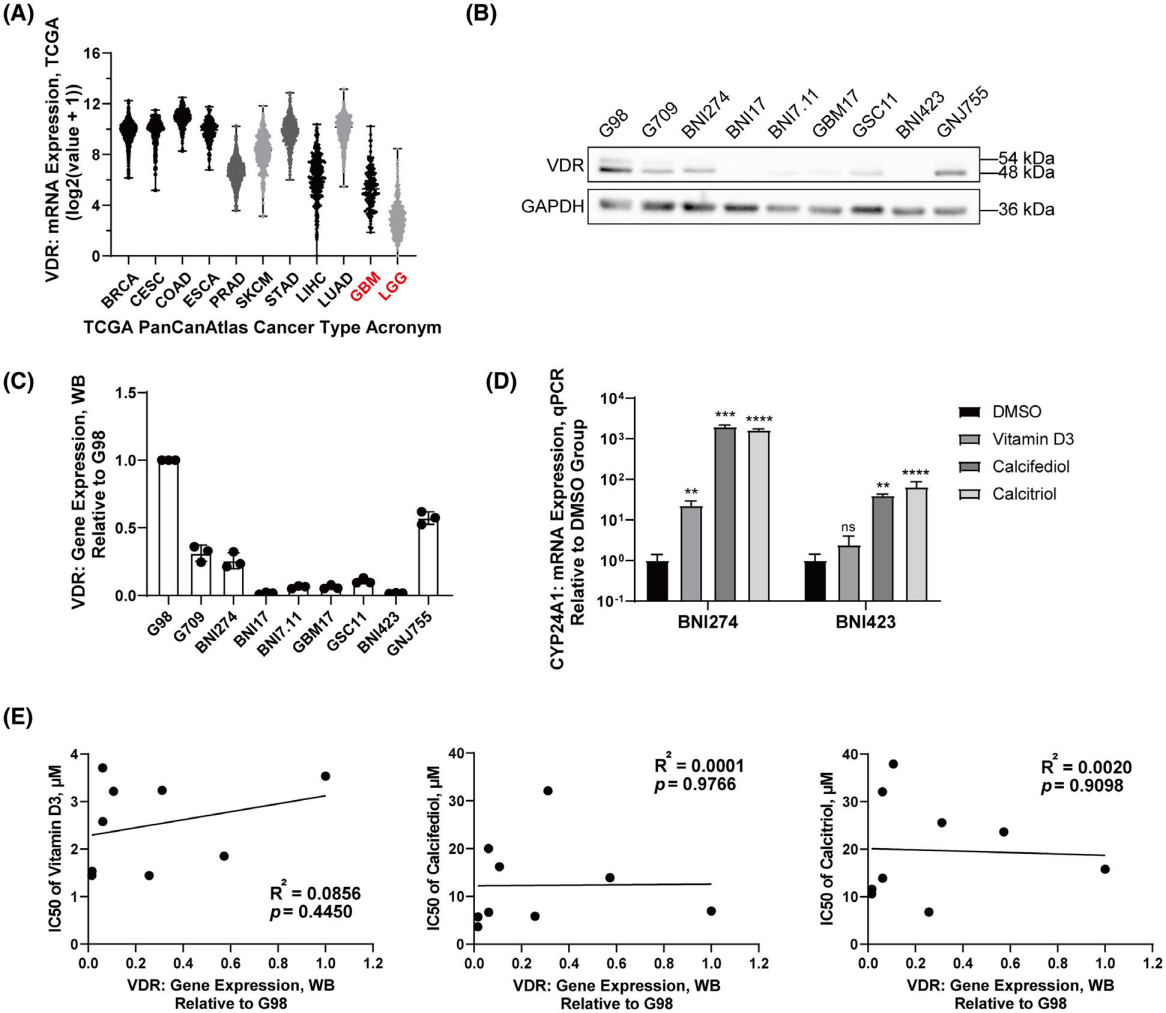
The correlation between the expression level of VDR in patient‐derived glioma cell lines and their sensitivity to vitamin D. (A) mRNA expression of *VDR* in nine common cancer types, low grade glioma and GBM from TCGA gene expression data. Median with range. BRCA, breast invasive carcinoma; CESC, cervical squamous cell carcinoma and endocervical adenocarcinoma; COAD, colon adenocarcinoma; ESCA, esophageal carcinoma; LIHC, liver hepatocellular carcinoma; LUAD, lung adenocarcinoma; PRAD, prostate adenocarcinoma; SKCM, skin cutaneous melanoma; STAD, stomach adenocarcinoma. (B, C) Protein expression levels of VDR by western blots with the indicated antibodies (B) and quantification of western blots (C) in patient‐derived glioma cell lines. (D) mRNA expression of *CYP24A1* in BNI274 and BNI423 after 30 h of treatment with vitamin D3, calcifediol or calcitriol by quantitative PCR. The concentration of vitamin D3, calcifediol or calcitriol used was 5 μm for BNI274 and 2.5 μm for BNI423. Data are the mean ± SD, *n* = 3, Student's *t*‐test: **P < 0.01, ***P < 0.001, ****P < 0.0001. ns, not significant. (E) The correlation between the IC_50_ of the compounds in patient‐derived glioma cell lines and their relative protein expression of VDR.

Before verifying the hypothesis that vitamin D3 does not obtain an inhibitory effect for patient‐derived glioma cell lines by activating VDR, it is necessary to confirm the expression level of *VDR* in glioma and patient‐derived glioma cell lines and the correlation between compounds and *VDR* signaling. Here, data on mRNA expression of *VDR* from The Cancer Genome Atlas (TCGA) (http://www.cancer.gov/tcga) are presented (Fig. 3A). Compared with other common cancers, gliomas, including GBM and low‐grade gliomas, had relatively low mRNA expression of *VDR*. Consistent with the TCGA data, the protein expression level of the VDR gene in most of patient‐derived glioma cell lines tested was also relatively lower (Fig. 3B,C). In particular, BNI423 and BNI17 almost did not express *VDR*. Then the expression level of *CYP24A1*, a key enzyme directly involved in vitamin D metabolism, was measured to confirm that these compounds, including vitamin D3, calcifediol, and calcitriol, could affect the *VDR* signaling in tested cell lines. The results showed that the mRNA expression of *CYP24A1* in patient‐derived glioma cell lines increased exponentially after treatment with these compounds (Fig. 3D). To explore whether there is a relationship between the sensitivity of cell lines to these compounds and their expression levels of *VDR*, the correlation between the IC_50_ of the compounds and the relative expression of VDR proteins was calculated. However, there was no strong or statistically significant correlation found between them (Fig. 3E).

**Fig. 4:**
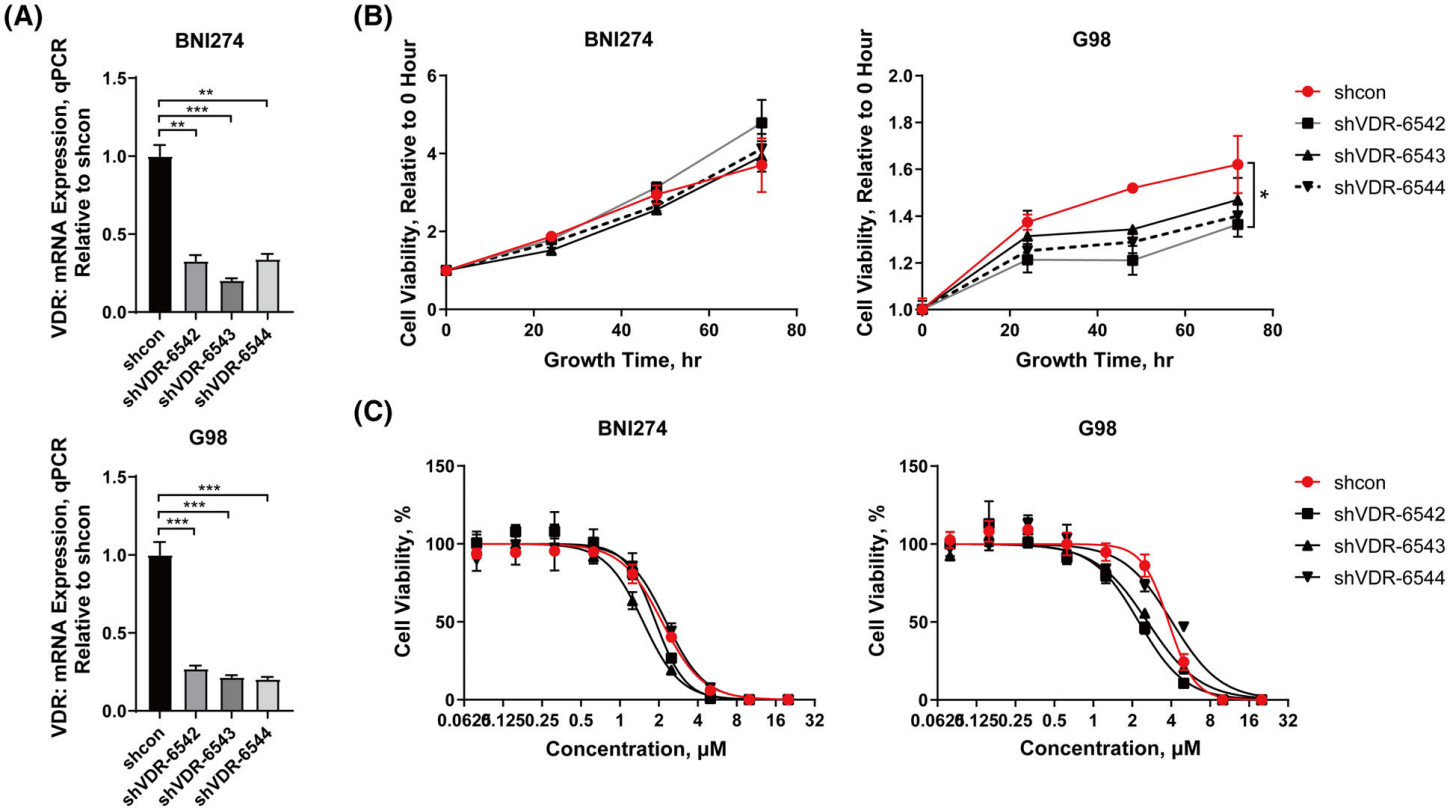
Gene silencing of *VDR* did not affect the sensitivity of patient‐derived glioma cell lines to vitamin D3. BNI274 and G98 were infected lentivirus with shRNA of VDR for *VDR*‐knockdown cell lines. mRNA expression of *VDR* (A) by quantitative PCR, cell growth curves (B) by cell viability assays and drug dose curves after exposure to vitamin D3 (C) by cell viability assays. Data are the mean ± SD, *n* = 3. Student's *t*‐test: *P < 0.05, **P < 0.01, and ***P < 0.001.

BNI274 and G98, which had relatively higher *VDR* expression levels in patient‐derived glioma cell lines, were selected for a *VDR*‐knockdown assay by shRNA. The knockdown efficiency of *VDR* is shown in Fig. 4A. The growth rate of BNI274 was almost unchanged after *VDR* knockdown, and only one *VDR* knockdown cell line of G98 had a significant decrease in the growth rate (*P* = 0.0291) (Fig. 4B). Then, the inhibitory effect of vitamin D3 on these *VDR* knockdown cell lines was compared with non‐knockdown cell lines. From the results of drug dose curves, the significant decrease in *VDR* expression did not affect the inhibition of cell viability by vitamin D3 (Fig. 4C).

**Fig. 5:**
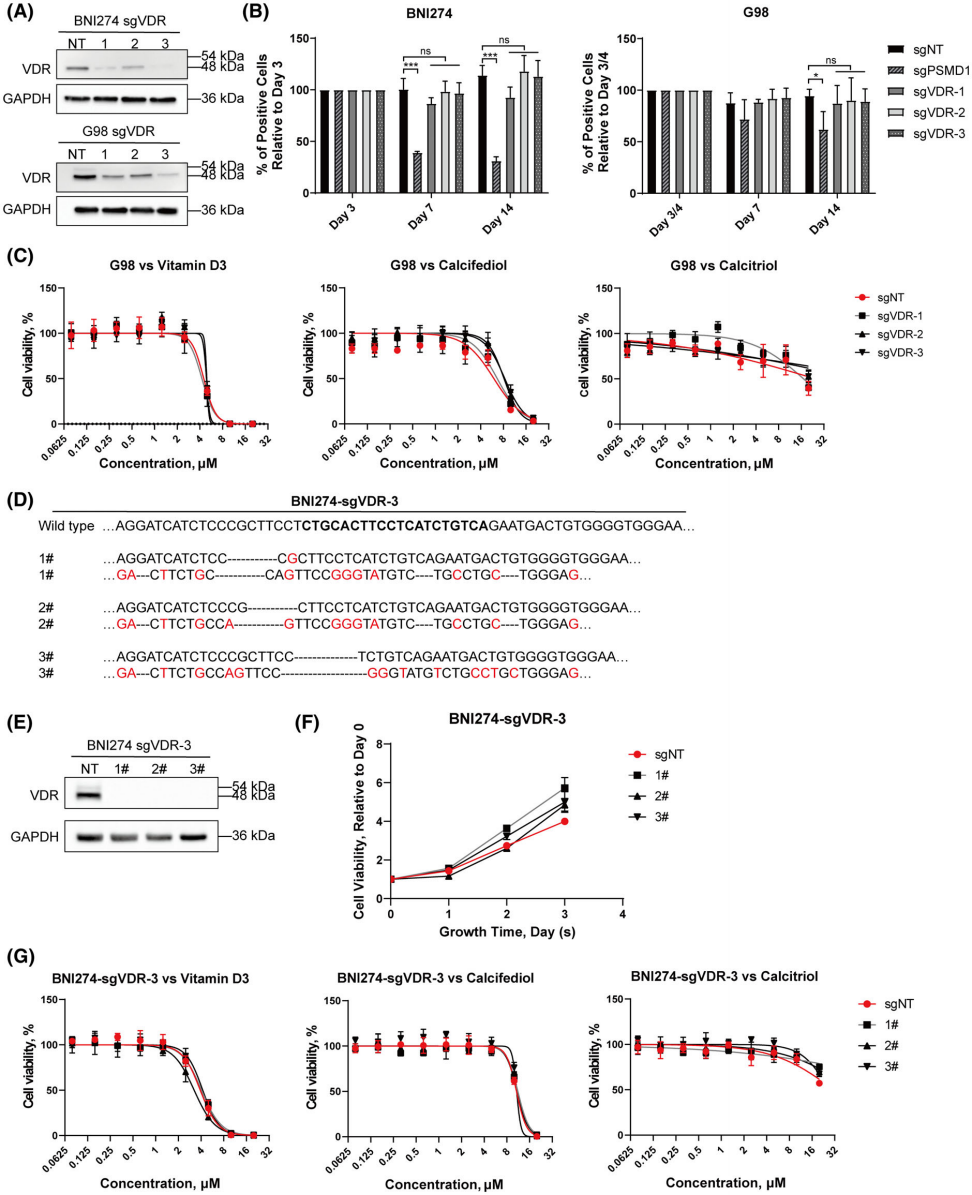
Gene knockout of *VDR* did not affect the sensitivity of patient‐derived glioma cell lines to vitamin D3. BNI274 and G98 were infected lentivirus with sgRNA of *VDR* for VDR‐knockout cell lines. (A) Assessment of knockout efficiency for VDR with western blots with the indicated antibodies in VDR‐knockout polyclonal cell lines. (B) Cell growth was analyzed by CRISPR competition assay in VDR‐knockout polyclonal cell lines. (C) Drug dose curves after exposure to vitamin D3, calcifediol or calcitriol by cell viability assays in VDR‐knockout polyclonal cell lines of G98. (D, E) Assessment of knockout efficiency for VDR with Sanger sequencing (D) and western blots (E) in VDR‐knockout monoclonal cell lines of BNI274. The target site is marked in bold in wild‐type and the mutated base is marked in red. (F) Cell growth curves by cell viability assays. (G) Drug dose curves after exposure to vitamin D3, calcifediol or calcitriol by cell viability assays in VDR‐knockout monoclonal cell lines of BNI274. Unless otherwise stated, data are reported as the mean ± SD, *n* = 3. Student's *t*‐test: *P < 0.05, ***P < 0.001. ns, not significant.

Because CRISPR/Cas9 technology may have the lower off‐target effect [40], we next used CRISPR/Cas9 technology to perform *VDR* knockout experiments to verify the experimental results of shRNA. The knockout efficiency of *VDR* in BNI274 and G98 is shown in Fig. 5A. Because the polyclonal cell lines used in Fig. 5A–C, the results of western blots showed that there retained a residual amount of VDR protein expression. To examine the long‐term effects of *VDR* knockout on the growth of cell lines, CRISPR competition assays were performed. The results showed that there was no statistically significant change in the growth rate of the *VDR*‐knockout cell lines (Fig. 5B). Here, *PSMD1* was used as the positive control affecting cell growth. Whether knocking out *VDR* in G98 altered their response to these compounds was also examined. After treatment using high concentrations, knockout of *VDR* gene expression altered the sensitivity of some knockout cell lines to calcitriol or calcifediol. However, the changes in the sensitivity of VDR‐knockout G98 to vitamin D3 were not found (Fig. 5C).

Monoclonal cell lines with the biallelic knockout of *VDR* gene were selected to repeat these experiments to avoid the influence of residual protein on the results. Polyclonal cell line BNI274‐sgVDR3, which had the stronger *VDR* knockout efficiency, was chosen to culture monoclonal cell lines by limiting dilution and flow sorting. The monoclonal cell lines were identified by Sanger sequencing and western blots to confirm their biallelic knockout of the *VDR* gene (Fig. 5D,E). Then, the growth rate of these cell lines was measured, and results still showed that the knockout of *VDR* gene did not inhibit the cell proliferation in tested cell lines (Fig. 5F). Furthermore, the knockout of *VDR* gene also did not change the sensitivity of BNI274 to vitamin D3 (Fig. 5G).

In summary, these results showed that the *VDR* was not an essential gene for patient‐derived glioma cell lines, nor did it affect the inhibitory effect of vitamin D3 on patient‐derived glioma cell lines. Such evidence supports our hypothesis that the inhibitory effects of vitamin D3 on patient‐derived glioma cell lines were not obtained through *VDR*.

### Vitamin D3 downregulated the cholesterol homeostasis pathway of patient‐derived glioma cell lines

**Fig. 6:**
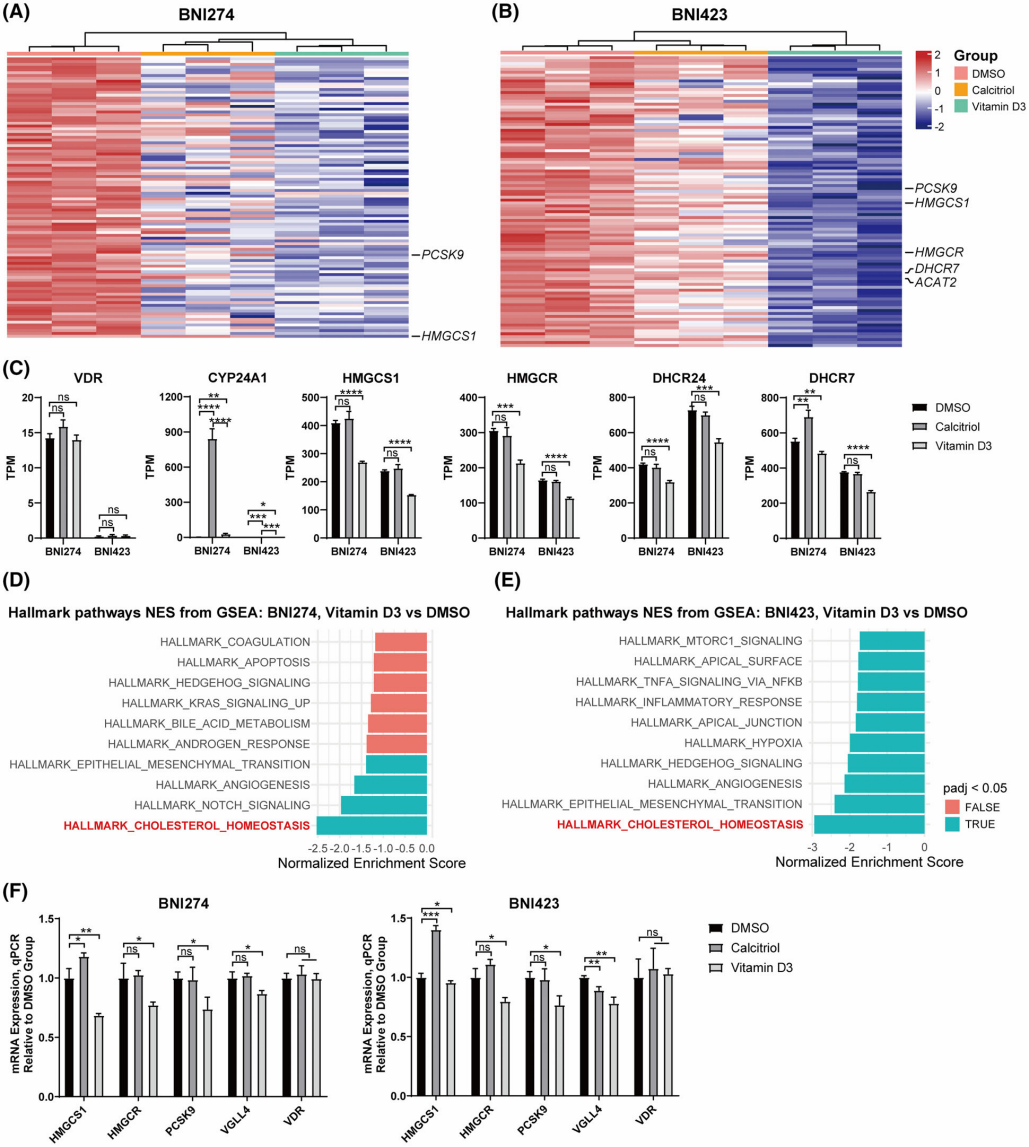
Cholesterol homeostasis pathways were significantly down‐regulated in patient‐derived glioma cell lines treated with vitamin D3. RNA sequencing analysis in BNI274 and BNI423 treated with calcitriol and vitamin D3 for 30 h. The concentration of vitamin D3 or calcitriol used was 5 μm for BNI274 and 2.5 μm for BNI423. (A, B) The top 100 genes down‐regulated by log_2_‐fold change in BNI274 (A) and BNI423 (B) between dimethylsulfoxide‐treated and vitamin D3‐treated are shown. The genes related to the cholesterol homeostasis pathway were annotated. ACAT2, acetyl‐CoA acetyltransferase 2. (C) The genes of interest are shown with TPM. TPM, transcripts per million. (D, E) Gene set enrichment analysis for the top 10 down‐regulated hallmark pathways in BNI274 (D) and BNI423 (E) between dimethylsulfoxide‐treated and vitamin D3‐treated are shown. (F) mRNA expression of genes related to cholesterol homeostasis in BNI274 and BNI423 after 30 h of treatment with calcitriol or vitamin D3 by quantitative PCR. DHCR24, 24‐dehydrocholesterol reductase; VGLL4, vestigial like family member 4. In quantitative PCR, data are the mean ± SD, *n* = 3, Student's *t*‐test: *P < 0.05, **P < 0.01, ***P < 0.001, ****P < 0.0001. ns, not significant.

The previous results excluded the effect of *VDR* in vitamin D3 treatment. To explore exactly how vitamin D3 affects patient‐derived glioma cell lines, RNA sequencing on vitamin D3‐treated cell lines was performed (Fig. 6A,B). Here, we selected BNI274 with *VDR* expression and BNI423 with almost no *VDR* expression (Fig. 3B) for experiments to avoid the influence of *VDR* on the results. The results of the expression of *VDR* and *CYP24A1* in RNA sequencing were consistent with the previous results (Fig. 6C). Notably, *CYP24A1* was significantly increased after calcitriol treatment of BNI274, which was different from vitamin D3 (Fig. 6C). This may indicate that not all vitamin D3 was converted to the active form in patient‐derived glioma cell lines, or that the rate of conversion was limited.

From gene set enrichment analysis for hallmark pathways, the cholesterol homeostasis pathway was found to be the most significantly down‐regulated pathway in both cell lines treated with vitamin D3 (Fig. 6D,E). The names of genes associated with cholesterol homeostasis are indictaed in Fig. 6A,B, and the expression levels of genes of cholesterol synthesis are shown separately in Fig. 6C. The results reveal that the down‐regulation of cholesterol homeostasis pathway was obtained in patient‐derived glioma cell lines treated with vitamin D3, but not calcitriol. The results were verified by a quantitative PCR, which was consistent with RNA sequencing (Fig. 6F).

### Vitamin D3 decreased the intracellular cholesterol content and cholesterol synthesis of patient‐derived glioma cell lines

The genes for which the expression level was most significantly decreased in the cholesterol homeostasis pathway after vitamin D3 treatment were proprotein convertase subtilisin/kexin type 9 (*PCSK9*), 3‐hydroxy‐3‐methylglutaryl‐CoA synthase 1 (*HMGCS1*), 3‐hydroxy‐3‐methylglutaryl‐CoA reductase (*HMGCR*), *DHCR7* and 24‐dehydrocholesterol reductase (*DHCR24*) (Fig. 6A–C). *HMGCS1*, *HMGCR*, *DHCR7* and *DHCR24* were the key genes associated with cholesterol synthesis [41]. *PCSK9* expression levels have also been reported to correlate with cholesterol levels [42]. Intracellular cholesterol levels correlate with the proliferation rate of some types of stem cells [43]. Therefore, we hypothesized that the inhibition of cell proliferation in patient‐derived glioma cell lines by vitamin D3 was a result of the inhibition of cholesterol synthesis.

**Fig. 7:**
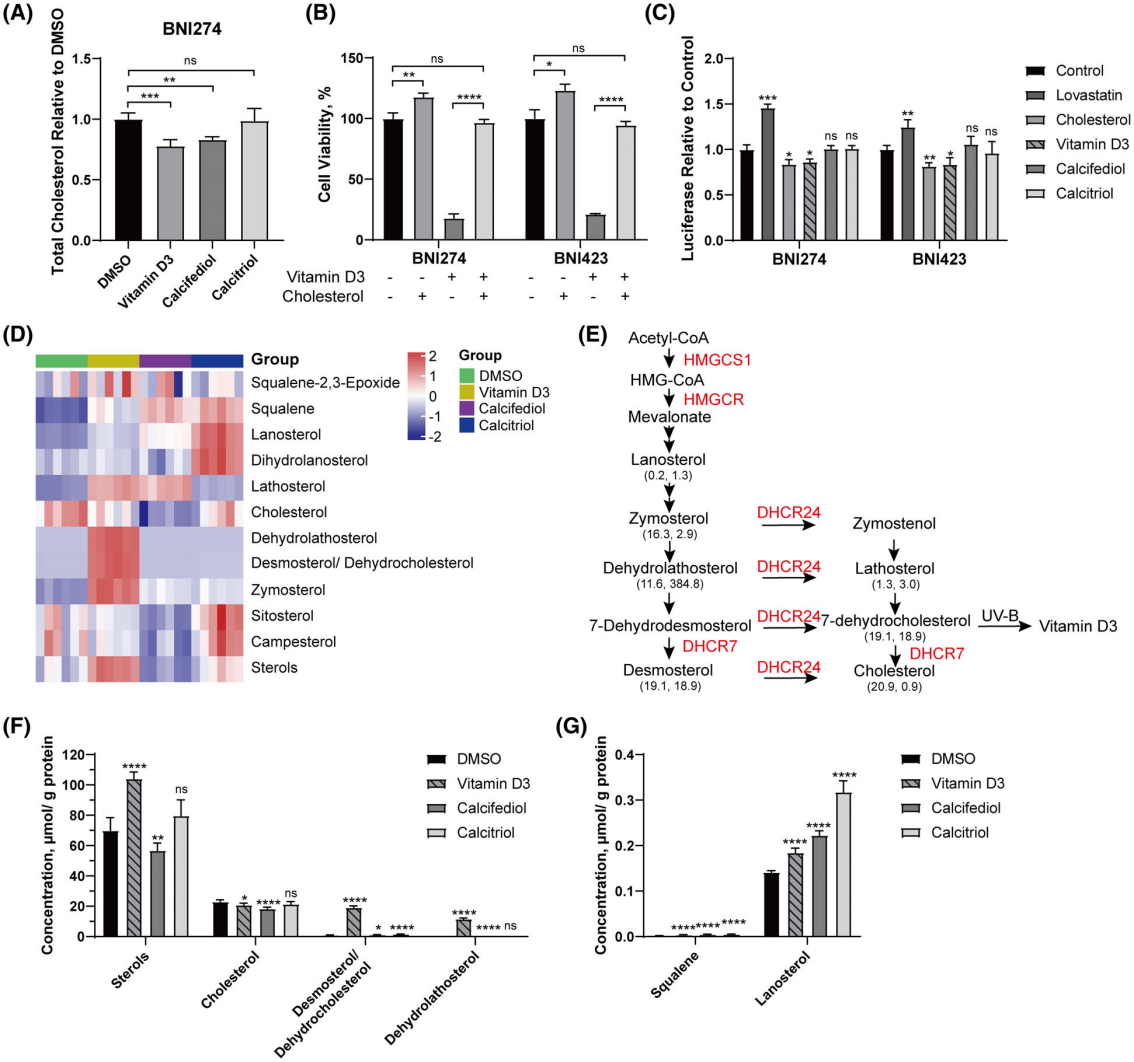
Vitamin D3 affected the cholesterol synthesis pathway in patient‐derived glioma cell lines. (A) Relative total cellular cholesterol of BNI274 after 24 h of treatment with vitamin D3, calcifediol or calcitriol was evaluated by a cholesterol detection assay; *n* = 3. (B) Cell viability assays for BNI274 and BNI423 after 72 h of incubation of vitamin D3 with or without 5 μm cholesterol; *n* = 3. (C) SRE‐reporter expression level of BNI274 or BNI423 after 24 h of treatment with vitamin D3, 4 μm cholesterol, 1 μm lovastatin, calcifediol or calcitriol by luciferase assays; *n* = 3. (D) Heatmap displaying the changes in cholesterol and other sterols in the cholesterol synthesis pathway after 24 h of treatment with vitamin D3, calcifediol, calcitriol or dimethylsulfoxide in BNI274; *n* = 6. (E) A simplified summary of cholesterol synthesis including sterol intermediates mainly affected by DHCR7 and DHCR24. The concentrations of sterols and sterol intermediates (left) in the vitamin D3‐treated group and the ratios to the control group (right) are shown. The unit of concentrations is μmol·g^−1^ protein. UV‐B, ultraviolet. (F, G) Concentrations of total sterols, cholesterol, desmosterol/dehydrocholesterol, dehydrolathosterol (F), squalene and lanosterol (G) in BNI274 were analyzed by LC/MS. Normalization was performed based on the total protein amount; *n* = 6. The concentration of vitamin D3, calcifediol or calcitriol used was 5 μm for BNI274 and 2.5 μm for BNI423. Data are reported as the mean ± SD. Student's t‐test: *P < 0.05, **P < 0.01, ***P < 0.001, ****P < 0.0001. ns, not significant.

Intracellular cholesterol levels in BNI274 treated with vitamin D3 were measured. Consistent with the hypothesis, total intracellular cholesterol did decrease compared to the control group (*P* = 0.0009) (Fig. 7A). When cholesterol was fed back into BNI274 and BNI423 treated with vitamin D3, their cell viability was significantly rescued and almost close to that of the control groups (Fig. 7B). Meanwhile, groups treated with cholesterol alone showed an increase in cell viability. This suggested that total cholesterol levels could affect the cell growth of patient‐derived glioma cell lines. At the same time, the decreased expression level of the *SRE* reporter suggested that the levels of total intracellular sterols may be significantly increased after vitamin D3 treatment (Fig. 7C) [44]. High intracellular sterols were reported to inhibit the regulation and activation of transcription of genes encoding many enzymes by sterol regulatory element binding protein, including important genes in cholesterol synthesis and *PCSK9* [45, 46, 47].

To further explore the changes in the contents of sterols after the treatment of vitamin D3, sterolomics analysis was performed by LC/MS. The results showed that the total sterols in patient‐derived glioma cell lines increased significantly after vitamin D3 treatment, whereas total cholesterol decreased (Fig.7D,F), which was consistent with the results shown in Fig.7A,C. More importantly, the concentrations of desmosterol and 7‐DHC (dehydrocholesterol) were increased by 18‐fold in the group treated with vitamin D3. In addition, the concentrations of dehydrolathosterol, lathosterol and zymosterol were also increased 383‐fold, two‐fold and two‐fold, respectively, compared to the control group. These significantly increased sterols are located at the end of cholesterol synthesis, and DHCR7 or/and DHCR24 are the key enzymes regulating these sterols (Fig. 7E). In the data from the RNA sequencing, the mRNA expression levels of DHCR7 and DHCR24 were decreased after vitamin D3 treatment (Fig. 6C). 7‐DHC and other sterol intermediates have been reported to be involved in the regulation of the inhibition of *HMGCR* mRNA expression or the degradation of HMGCR protein, thereby regulating the cholesterol synthesis pathway [48, 49, 50].

In summary, vitamin D3 stagnated cholesterol synthesis by inhibiting the expression levels of *DHCR7* and *DHCR24*, which led to excessive accumulation of downstream sterol intermediates. The accumulation of sterol intermediates further strengthened the inhibition of the cholesterol synthesis and cholesterol homeostasis pathways. This was the main mechanism by which vitamin D3 inhibited cell viability and proliferation in patient‐derived glioma cell lines.

## Discussion

Changes in genetic and epigenetic signaling pathways in tumors often promote metabolic reprogramming [50]. Altered cellular metabolism is one of the hallmarks of glioma [51]. Significant increases in lipid levels and lipid metabolism have been reported in glioma [53, 54]. In addition, the brain is the organ with the highest cholesterol level in the body, with approximately 20% of total cholesterol [54]. In addition, because of the blood–brain barrier, almost all cholesterol in the brain is synthesized *de novo* by astrocytes [55]. Meanwhile, essential fatty acids that can cross the blood–brain barrier are important raw materials for glioma to synthesize lipids [51]. Therefore, cholesterol uptake and lipid synthesis are important for glioma. Lipid metabolism is a potential therapeutic target for glioma.

Although the research on vitamin D in GBM has focused on the active form of vitamin D, in the present study, we demonstrated that patient‐derived glioma cell lines were more sensitive to vitamin D3 than calcitriol and calcifediol. Interestingly, vitamin D3 obtained more significant inhibitory effects on cell viability and cell proliferation of patient‐derived glioma cell lines by inhibiting the expression levels of *DHCR7* and *DHCR24*, thereby inhibiting cholesterol synthesis and cholesterol homeostasis pathways. In our view, the effects of vitamin D3 on cholesterol homeostasis have been rarely reported, and its mechanisms have scarcely been studied [57, 58]. Meanwhile, vitamin D3 shares the same precursor 7‐DHC with cholesterol, and DHCR7 plays a key enzyme involved in the conversion of 7‐DHC to cholesterol. In addition, vitamin D3, but not its active form, has been reported to rapidly inhibit the activity of DHCR7 in keratinocytes [58]. As one of the downstream products of 7‐DHC, vitamin D3 was involved in the regulation of DHCR7 expression level and/or enzyme activity. The effect of vitamin D3 on cholesterol homeostasis is not direct but, instead, is suggested to be a result of excessive accumulation of sterol intermediates because of the inhibition of DHCR7.

**Fig. 8:**
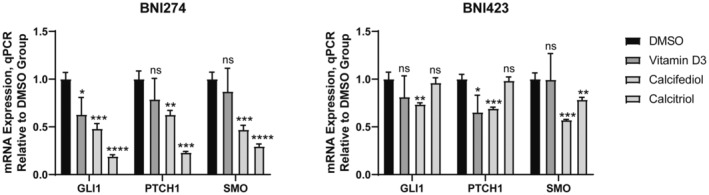
Vitamin D had differential effects on Hedgehog signaling in different patient‐derived glioma cell lines. mRNA expression of genes related to Hedgehog signaling in BNI274 and BNI423 after 30 h of treatment with vitamin D3, calcifediol or calcitriol by quantitative PCR. GLI1, GLI family zinc finger 1; PTCH1, Patched 1; and SMO, Smoothened. The concentration of vitamin D3, calcifediol or calcitriol used was 5 μm for BNI274 and 2.5 μm for BNI423. Data are the mean ± SD, *n* = 3. Student's *t*‐test: *P < 0.05, **P < 0.01, ***P < 0.001, ****P < 0.0001. ns, not significant.

It is worth noting that mutations in gene *DHCR7* are associated with a disease called Smith–Lemly–Opitz‐syndrome (SLOS), which defects in hedgehog signaling [60, 61]. Furthermore, vitamin D3 has been reported to regulate hedgehog signaling and inhibit cell proliferation by antagonizing Smoothened (*SMO*) and inhibiting the expression level of GLI family zinc finger 1 (*GLI1*) in some types of cell lines [37, 62]. Such evidence made the assessment of key genes in the hedgehog pathway necessary. The mRNA expression levels of Patched 1 (*PTCH1*), *SMO* and *GLI1* were measured by a quantitative PCR (Fig. 8). The results showed that the effect of vitamin D3 on these genes is weak compared to calcitriol or calcifediol. Especially on BNI274, calcitriol exhibited strong inhibition of hedgehog signaling. However, in contrast, vitamin D3 had a more significant inhibitory effect on the cell viability and cell proliferation of BNI274 than calcitriol (Table 1). At the same time, the inhibitory effect of calcitriol on these genes with BNI423 was limited compared to that of BNI274. Therefore, hedgehog signaling was one of the pathways affected by vitamin D3 in patient‐derived glioma cell lines, but not the most pivotal pathway. Moreover, this regulation was cell‐specific.

A major limitation of the present is that the experimental model used was only cell line, but the environment *in vivo* is more complex. In addition, whether this regulation occurred only on patient‐derived glioma cell lines or is a general feedback mechanism is also an important question. Limited by the lack of instruments, we were not able to examine whether the enzymatic activity of DHCR7 was inhibited after vitamin D3 treatment as previously reported, and only confirmed that the mRNA expression level of *DHCR7* was decreased. How vitamin D3 regulated the mRNA expression or/and enzyme activity of DHCR7 at the microscopic level is still a very interesting question. These questions are worthy of further study.

Although our works focused on vitamin D3, calcifediol and calcitriol also had interesting phenomena. Sterolomics in vitamin D3‐, calcifediol‐ or calcitriol‐treated groups was significantly different from each other (Fig. 7D). In the calcifediol‐treated group, the concentration of total sterols and total cholesterol was decreased and the concentration of squalene and lathosterol was increased (Fig. 7F,G). In the calcitriol‐treated group, the sterol intermediates accumulated mainly were squalene, lanosterol and dihydrolanosterol, which are located upstream of cholesterol synthesis (Fig. 7G). Although the accumulated lanosterol was reported to induce rapid degradation of HMGCR [47], the concentration of lanosterol in the calcitriol‐treated group increased slightly compared to the excessive accumulation of sterol intermediates in the vitamin D3‐treated group, which may explain why the cholesterol synthesis was not inhibited after treatment with calcitriol. Because calcitriol has been more commonly reported and investigated for its effects in tumors, how calcifediol and calcitriol regulate the cholesterol synthesis pathway is worthy of further study.

## Conflicts of interest

The authors declare that they have no conflicts of interest.

### Peer review

The peer review history for this article is available at https://www.webofscience.com/api/gateway/wos/peer‐review/10.1002/2211‐5463.13679.

## Author contributions

RY and JC conceptualized and designed the study. RY wrote the manuscript. WZ, Y‐PY, GC, ZG and XW provided the human glioma cell lines. All authors read and approved the final version of the manuscript submitted for publication.

## Data Availability

RNA sequencing data are available through Genome Sequence Archive for Human under accession HRA003356. Other data used to support the findings of the present study are available from the corresponding author upon reasonable request.
